# Early therapeutic plasma exchange in septic shock: a prospective open-label nonrandomized pilot study focusing on safety, hemodynamics, vascular barrier function, and biologic markers

**DOI:** 10.1186/s13054-018-2220-9

**Published:** 2018-10-30

**Authors:** Hannah Knaup, Klaus Stahl, Bernhard M. W. Schmidt, Temitayo O. Idowu, Markus Busch, Olaf Wiesner, Tobias Welte, Hermann Haller, Jan T. Kielstein, Marius M. Hoeper, Sascha David

**Affiliations:** 1 Division of Nephrology and Hypertension, Hannover Medical School, Carl-Neuberg-Str.1, 30625 Hannover, Germany; 2 Department of Gastroenterology, Hepatology and Endocrinology, Hannover Medical School, Hannover, Germany; 3 Department of Respiratory Medicine and German Centre of Lung Research (DZL), Hannover Medical School, Hannover, Germany; 4 Medical Clinic V, Nephrology, Rheumatology, Blood Purification, Academic Teaching Hospital Brunswick, Braunschweig, Germany

**Keywords:** Extracorporeal treatment, Plasmapheresis, Endothelium, Blood purification, Fresh frozen plasma

## Abstract

**Background:**

Given the pathophysiological key role of the host response to an infection rather than the infection per se, an ideal therapeutic strategy would also target this response. This study was designed to demonstrate safety and feasibility of early therapeutic plasma exchange (TPE) in severely ill individuals with septic shock.

**Methods:**

This was a prospective single center, open-label, nonrandomized pilot study enrolling 20 patients with early septic shock (onset < 12 h) requiring high doses of norepinephrine (NE; > 0.4 μg/kg/min) out of 231 screened septic patients. Clinical and biochemical data were obtained before and after TPE. Plasma samples were taken for ex-vivo stimulation of human umbilical vein endothelial cells (HUVECs) to analyze barrier function (immunocytochemistry and transendothelial electrical resistance (TER)). Cytokines were measured by cytometric bead array (CBA) and enzyme-linked immunosorbent assays (ELISAs). An immediate response was defined as > 20% NE reduction from baseline to the end of TPE.

**Results:**

TPE was well tolerated without the occurrence of any adverse events and was associated with a rapid reduction in NE (0.82 (0.61–1.17) vs. 0.56 (0.41–0.78) μg/kg/min, *p* = 0.002) to maintain mean arterial pressure (MAP) above 65 mmHg. The observed 28-day mortality was 65%. Key proinflammatory cytokines and permeability factors (e.g., interleukin (IL)-6, IL-1b, and angiopoietin-2) were significantly reduced after TPE, while the protective antipermeability factor angiopoietin-1 was not changed. Ex-vivo stimulation of HUVECs with plasma obtained before TPE induced substantial cellular hyperpermeability, which was completely abolished with plasma obtained after TPE.

**Conclusions:**

Inclusion of early septic shock patients with high doses of vasopressors was feasible and TPE was safe. Rapid hemodynamic improvement and favorable changes in the cytokine profile in patients with septic shock were observed. It has yet to be determined whether early TPE also improves outcomes in this patient cohort. An appropriately powered multicenter randomized controlled trial is desirable.

**Trial registration:**

Clinicaltrials.gov, NCT03065751. Retrospectively registered on 28 February 2017.

**Electronic supplementary material:**

The online version of this article (10.1186/s13054-018-2220-9) contains supplementary material, which is available to authorized users.

## Background

Sepsis is defined as life-threatening organ dysfunction caused by a dysregulated host response to infection; if hypotension is refractory to volume resuscitation and serum lactate is elevated it is termed septic shock [1]. In the absence of a specific intervention other than anti-infective drugs, mortality rates can still be as high as 60% [2]. The overwhelming host response is a key driver of morbidity and mortality [3]. Despite our increasing understanding of the molecular and pathophysiological processes underlying sepsis-associated organ injury, treatment options are all nonspecific with regard to the host response [4]. There is an unmet need to improve our therapeutic strategies by directly targeting and modulating the pathological response of the host. Part of the failure to develop effective strategies might be attributable to the complexity and nonlinearity of sepsis pathophysiology making it unlikely for a single specific agent to successfully influence the host response in its whole nature [5, 6].

The theoretical concept of therapeutic plasma exchange (TPE) in sepsis combines two major aspects in one intervention: 1) removal of harmful circulating molecules (as part of the injurious cytokine storm) that directly contribute to the manifestation of the disease; and 2) replacement of protective plasma proteins that compensate for the loss of factors important for coagulation (e.g., activated protein C, antithrombin, tissue factor pathway inhibitor), fibrinolysis (e.g., von Willebrand factor (vWF) cleaving proteases), and that counteract inflammation and vascular leakage (e.g., angiopoietin-1, vascular endothelial growth factor (VEGF)) to ultimately restore hemostasis [7].

So far, the available data on TPE in sepsis are poor compared with other blood purification techniques (summarized in [8]); mostly, case reports (e.g., [9]) and uncontrolled retrospective studies [10, 11] have been published. A recent meta-analysis found only two single-center randomized controlled trials (RCTs) in adults in which a reduced mortality (risk ratio (RR) 0.63, 95% confidence interval (CI) 0.42 to 0.96) was reported [12]. The largest RCT showed a promising trend towards improved survival [13]. The American Society for Apheresis (ASFA) stated in their 2016 guidelines “the optimum role of apheresis therapy is not established; decision making should be individualized”, and gave a weak recommendation [14].

We hypothesized that TPE as an additive treatment might modulate the deleterious host response in a comprehensive approach affecting hemodynamics, fluid balances, vascular barrier function, and cytokine profiles in the most critical septic shock patients if applied at the earliest possible time point. Therefore, we designed this exploratory study to demonstrate the safety and feasibility with regard to recruitment and enrollment for a larger RCT, and to secondarily test preliminary efficacy with regard to the abovementioned hemodynamics and biochemical markers.

## Methods

### Study population

**Fig. 1:**
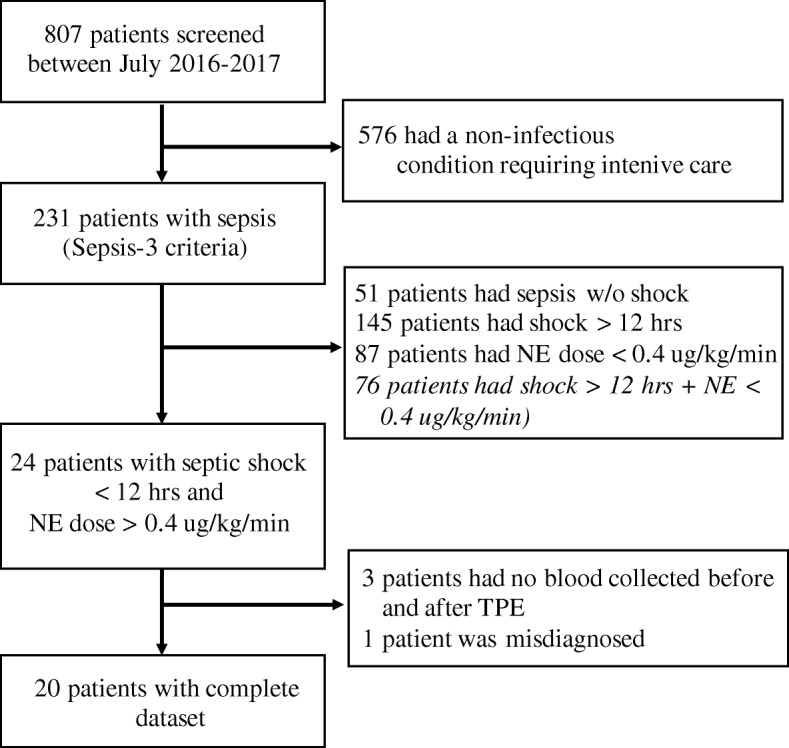
Flow chart of study participants. NE norepinephrine, TPE therapeutic plasma exchange

This was a prospective single-center, open-label, nonrandomized pilot study. We screened 807 patients submitted to our 14-bed medical intensive care unit (ICU) from July 2016 to July 2017 for the presence of sepsis as per the SEPSIS-3 definition [1] (Fig. 1). All patients were treated according to the 2012 Surviving Sepsis Campaign (SSC) guidelines [15]. The ethics committee of Hannover Medical School approved the protocol (no. 2786–2015), and written informed consent was obtained from participants or authorized representatives. The study was performed in accordance with the ethical standards laid down in the 1964 Declaration of Helsinki and its later amendments.

#### Inclusion and exclusion criteria

Patients were included based on: 1) septic shock with the need for vasopressors < 12 h prior to entry; and 2) profound systemic hypotension requiring norepinephrine (NE) doses of > 0.4 μg/kg/min despite adequate intravenous fluid resuscitation (≥ 30 mL/kg bodyweight crystalloids). TPE had to be initiated within 6 h after study inclusion. For exclusion criteria, we used pregnancy or breast feeding, age < 18 years, end-stage chronic disease, and the presence of a directive to withhold life-sustaining treatment.

#### Therapeutic plasma exchange (TPE)

Vascular access was established by venous insertion of an 11-French two-lumen hemodialysis catheter. Based on previous experience we decided to use one TPE since hemodynamic improvements were only achieved by the very first exchange (data not shown). TPE was performed against fresh frozen plasma (FFP), exchanging 1.2× the individually calculated plasma volume with a blood flow of 60 (55–63) mL/min. Anticoagulation during TPE was achieved by regional citrate infusion. In patients with acute kidney injury, dialysis was interrupted for the duration of TPE (110 (93–120) min). Blood samples were drawn immediately before and after TPE. Patients were closely followed for the next 28 days and survival was recorded. NE dose was titrated every 10–15 min to achieve a mean arterial pressure (MAP) above 65 mmHg.

The primary aim of this study was to evaluate the safety and feasibility with regard to recruitment and enrollment within 12 h of shock for a planned RCT (clinicaltrials.gov NCT03065751) that would allow us to investigate hard clinical endpoints.

In addition, preliminary efficacy was evaluated by longitudinal assessment before and after TPE for:

- NE dose to maintain MAP ≥ 65 mmHg
- MAP
- 6-h fluid balances
- C-reactive protein (CRP), procalcitonin (PCT), white blood cell (WBC) counts
- International normalized ratio (INR), platelets
- cytokines (interleukin (IL)-6, IL-1b, IL-8, and IL-10)
- permeability-regulating factors (angiopoietin-1, -2, and soluble receptor of tyrosine kinase with immunoglobulin-like and EGF-like domains 2 (sTie2))
- preload (stroke volume variance (SVV) and global end-diastolic volume index (GEDI))
- afterload (systemic vascular resistance index (SVRI))
- cardiac index

Furthermore, 28-day survival was analyzed for the whole cohort and for subgroups (immediate and sustained responders); immediate response to TPE was defined as a reduction of the NE > 20% from baseline immediately following completion of TPE, and sustained response was described as any reduction of Sequential Organ Failure Assessment (SOFA) score within 48 h post-TPE, as described previously [16].

### Endothelial ex-vivo stimulation with plasma from septic shock patients

We used human umbilical vein endothelial cells (HUVECs) that were isolated from umbilical vein donors (ethical improvement no. 1303–2012) and cultured as described previously [17]. To mimic the septic vascular phenotype in confluent HUVEC monolayers, their growth medium was supplemented with 5% plasma obtained from septic patients within minutes before and after TPE.

#### Fluorescent immunocytochemistry

Thirty minutes after treatment with patient plasma, cells were fixed in 2.5% paraformaldehyde, permeabilized, and incubated with primary antibody (VE-cadherin; BD Bioscience, San Diego, CA), followed by with secondary Alexa-antibody and phalloidin [18].

#### Transendothelial electrical resistance (TER)

To quantify endothelial permeability, serial TERs were recorded with an electric cell-substrate impedance sensing (ECIS) approach in triplicate (Ibidi, Applied BioPhysics Inc.) as described previously [19].

#### Measurement of circulating cytokines and permeability factors

Angiopoietin-1, -2, and sTie2 were measured by enzyme-linked immunosorbent assays (ELISAs; R&D systems, Minneapolis) and a panel of cytokines was assessed by cytometric bead array (CBA; BD Bioscience) on a fluorescence-activated cell sorting (FACS) platform.

### Statistical analysis

Date are presented as median (25% to 75% interquartile range (IQR)). Two-tailed *p* values of less than 0.05 were considered to indicate statistical significance. Paired *t* test or Wilcoxon test (for non-normally distributed variables) was utilized to compare longitudinal values before (pre-) and after (post-) TPE. Survival data were analyzed by log-rank test and visualized by Kaplan-Meier curves. We compared the subgroups of responders and nonresponders utilizing a Mann-Whitney *U* test for nominal variables and performing a χ^2^ test for categorical variables. We used GraphPad Prism 7 (La Jolla, CA) and SPSS Statistics (IBM) for data analysis and graph generation.

## Results

### Cohort characterization

Demographic and clinical details are summarized in Table 1. Sixty-five percent of the patients were men, and the median age was 52 (30–58) years. The lungs and the abdomen were the most common sites of infection. A causative pathogen was identified in 75% of the cases. All patients were treated with a combination of broad-spectrum antibiotics. Retrospectively, 95% of the initial treatment strategies were sensitive to the later identified microbial. Patient 9 had a positive blood culture for *Candida* that was not covered initially (Additional file 1: Table S1). Immediately after TPE was performed, all patients received an additional full dose of antibiotics.

**Table 1:** Demographic and clinical characteristics at baseline

Characteristic	Value
Age (years)	52 (30–58)
Sex (male/female), *n* (%)	13/7 (65/35)
Weight (kg)	85 (71–103)
Height (m)	1.79 (1.7–1.85)
BMI (kg/m^2^)	26.9 (22.2–31.9)
Sepsis onset, *n* (%)	
Community-acquired	10 (50)
Hospital-acquired	10 (50)
Site of infection, *n* (%)	
Lung	11 (55)
Abdomen	3 (15)
Urogenital	1 (5)
Soft tissue	3 (15)
Endocarditis	1 (5)
Mixed	1 (5)
Pathogen, *n* (%)	
Gram-positive	3 (15)
Gram-negative	5 (25)
Fungi	1 (5)
Mixed	5 (25)
Not identified	6 (30)
APACHE II	40.5 (35–46)
SOFA	18 (16–20)
ADAMTS13 (%)	44 (29–56.5)
Norepinephrine dose (μg/kg/min)	0.82 (0.61–1.17)
Mechanical ventilation, *n* (%)	19 (95)
Oxygenation index (PaO_2_/FiO_2_)	132 (96–229)
Renal replacement therapy, *n* (%)	13 (65)
Organ failure, *n* (%)	
Respiratory	19 (95)
Coagulation	14 (70)
Liver	10 (50)
Cardiovascular	20 (100)
Neurological	19 (95)
Renal	16 (80)
Multi organ failure, *n* (%)	
Two	0 (0)
Three	1 (5)
Four	6 (30)
Five	7 (35)
Six	6 (30)
Immunosuppression, *n* (%)	13 (65)

Values are shown as median (interquartile range) unless otherwise indicated

*ADAMTS13* A disintegrin and metalloprotease with thrombospondin-1-like domains 13, *APACHE* Acute Physiology and Chronic Health Evaluation, *BMI* body mass index, *SOFA* Sequential Organ Failure Assessment

Median (IQR) Acute Physiology and Chronic Health Evaluation (APACHE) II and SOFA scores were 40.5 (35.0–46.0) and 18 (16–20), respectively. Ninety-five percent of patients were mechanically ventilated and had an oxygenation index of 132 (96–229). Patients had at least three failed organ systems, while organ failure was defined as an organ-specific SOFA score of equal or more than 2. Acute kidney injury (AKI) with the need for renal replacement therapy (RRT) was present in 65% of the patients at inclusion.

### Feasibility and safety

Based on the inclusion criteria that aimed at identifying the sickest patients (NE > 0.4 μg/kg/min) at a very early state (shock < 12 h) we included 24 out of 231 sepsis patients within 1 year (Fig. 1). We were able to perform TPE within 6 h after inclusion. We did not obtain complete plasma samples from four patients, so these were excluded. The TPE procedure was found to be safe. Earlier reported side effects such as hypotension and allergic reactions [12] were not observed in this study. Given the successful recruitment and safety in this pilot study, a multicenter RCT investigating TPE in septic shock with a hard primary endpoint appears feasible.

### Immediate effects of TPE on clinical parameters

**Fig. 2:**
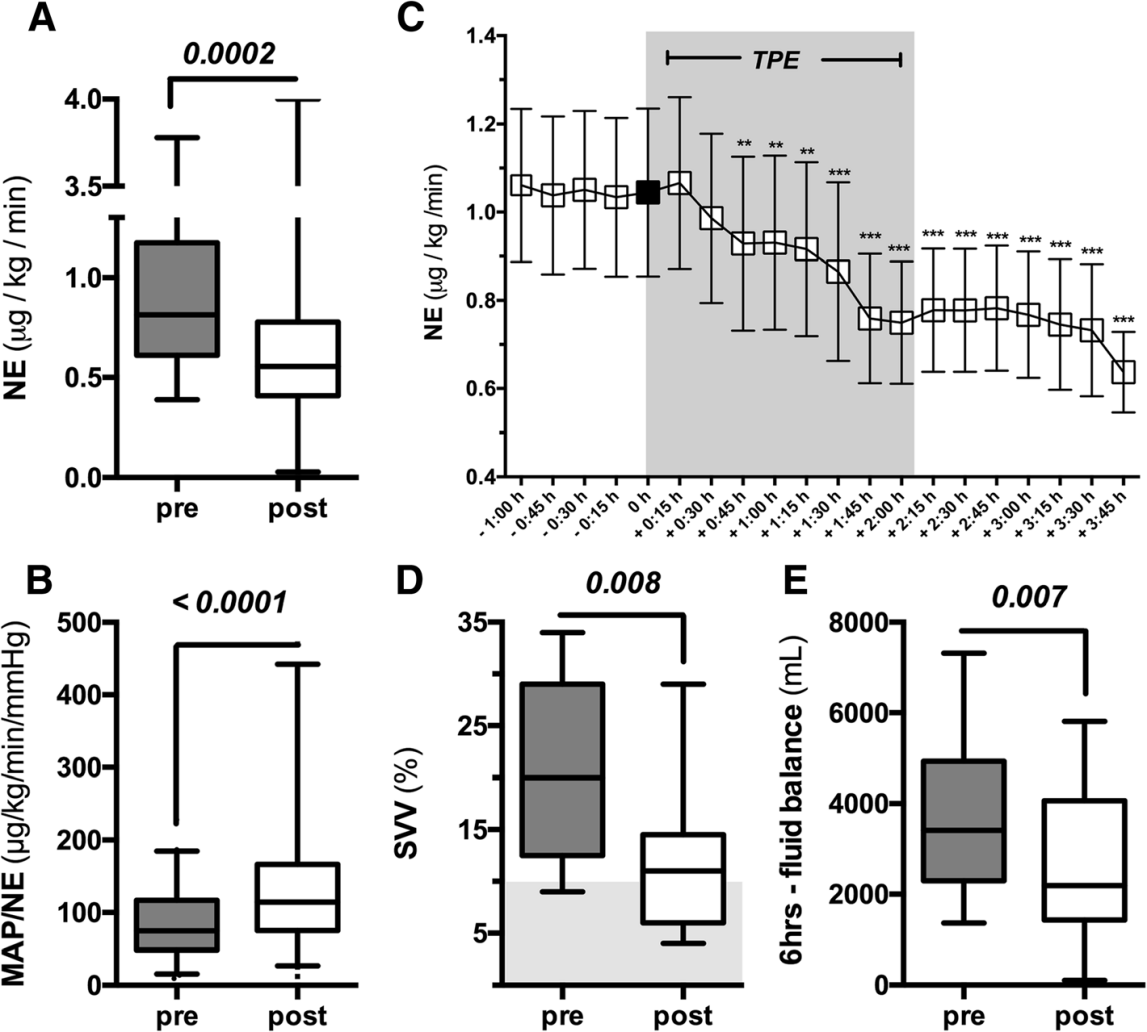
Hemodynamic improvements upon TPE. Box and whisker blots showing **a** the dose of norepinephrine (NE; μg/kg/min) immediately before the start of plasma exchange (pre) and after TPE (post) (*p* = 0.0002), and **b** the ratio of mean arterial pressure (MAP) over NE dose (*p* < 0.0001). **c** Peri-interventional (−60 to +105 min) longitudinal course of NE doses over the therapeutic plasma exchange (TPE) procedure assessed every 15 min (**p < 0.001, ***p < 0.0001, compared with time-point 0 highlighted in black). **d** Box and whisker blot of stroke volume variance (SVV) as a dynamic preload surrogate. Grey area highlights the reference range for healthy individuals (*p* = 0.008). **e** Box and whisker blot for fluid requirements 6 h before (pre) plasma exchange and 6 h after (post) TPE (*p* = 0.007)

The dose of NE after a single TPE was significantly reduced (pre-TPE 0.82 (0.61–1.17) vs. post-TPE 0.56 (0.41–0.78) μg/kg/min, *p* = 0.0002; Fig. 2a). The MAP/NE ratios before and after TPE were 74.9 (48.5–116.8) and 114.3 (75.3–166.7) μg/kg/min/mmHg (*p* < 0.0001; Fig. 2b), respectively. The longitudinal time course of NE doses during TPE is shown in Fig. 2c.

A subgroup of 10 patients had hemodynamic assessments performed by thermodilution (PiCCO®, Pulsion) (Additional file 2: Figure S1). Here, we observed a mild, but nonsignificant increase in cardiac index (2.85 (2.39–4.32) vs. 3.42 (2.71–5.19) L/min/m^2^, *p* = 0.375) which was not attributable to an increased heart rate (111 (91–126) vs. 104 (87–119) beats/min, *p* = 0.107). Afterload as assessed by SVRI was not changed (1450 (980–1873) vs. 1520 (1060–2126) dyn/s/cm^5^/m^2^, *p* = 0.695), while SVV improved significantly (20 (12.5–29)% vs. 11 (6–14.5)%, *p* = 0.008; Fig. 2d). Fluid intake could be limited compared with a 6-h period before TPE (3411 (2295–4933) vs. 2190 (1431–4060) mL, *p* = 0.007; Fig. 2e). Clinical and biochemical changes are summarized in Table 2.

**Table 2:** Changes in clinical and biochemical parameters after TPE

Variable	Therapeutic plasma exchange (TPE)		*p* value
	Before	After	
Clinical parameters			
MAP (mmHg)	65.5 (54.5–75.3)	69 (64–79.3)	0.07
NE dose (μg/kg/min)	0.82 (0.61–1.17)	0.56 (0.41–0.78)	0.0002*
MAP/NE (mmHg/μg/kg/min)	74.9 (48.5–116.8)	114.3 (75.3–166.7)	< 0.0001*
HR (bpm)	110.5 (91.3–125.5)	103.5 (86.8–119)	0.11
SVV (%)	20 (12.5–29)	11 (6–14.5)	0.008*
SVRI (dyne/s/cm^5^/m^2^)	1450 (980–1873)	1520 (1060-2126)	0.67
SVRI/NE (dyne/s/cm^5^/m^2^)/(μg/kg/min)	1743 (1008-2921)	2547 (1213-3923)	0.06
EVLWI (mL/kg)	14 (8–17)	11.5 (8–16.5)	0.93
GEDI (mL/m^2^)	670 (483–909)	755 (622–998)	0.12
Cardiac index (L/min/m^2^)	2.85 (2.39–4.32)	3.42 (2.71–5.19)	0.39
Fluid balance/6 h (mL)	3411 (2295-4933)	2190 (1431-4060)	0.007*
Gas exchange			
Oxygenation index (PaO_2_/FiO_2_)	132 (96–229)	115 (102–212)	0.94
AaDO_2_ (mmHg)	360 (251–541)	329 (247–489)	0.28
Inflammatory biomarkers			
CRP (mg/L)	236 (147–302)	174 (86–288)	0.07
PCT (ng/mL)	24.1 (16.9–83.7)	31 (14.8–87.3)	0.86
WBC (1/nL)	11.2 (0.93–34.8)	8.4 (1.2–25.6)	0.73
PLT (1/nL)	43.0 (16.8–112)	34.0 (20–66)	0.11
INR	1.76 (1.44–2.1)	1.43 (1.26–2.1)	0.16
Acid base balance			
pH	7.28 (7.19–7.34)	7.33 (7.23–7.38)	0.01*
pCO_2_ (mmol/L)	44.5 (35.3–56.3)	46 (37–55)	0.99
HCO_3_^−^ (mmol/L)	20.0 (17–23.8)	22.0 (20–24.7)	0.001*
Lactate (mmol/L)	6.5 (2.8–11.3)	6.5 (3.2–10.8)	0.84
Cytokines			
IL-8 (ng/mL)	1.35 (0.6–10.81)	1.09 (0.4–7.1)	0.009*
IL-1b (pg/mL)	147.1 (57.1–241.6)	92.2 (42.9–184.8)	0.01*
IL-6 (ng/mL)	10.8 (2.54–27.6)	4.6 (0.9–13.7)	0.005*
IL-10 (pg/mL)	143.3 (65.5–259.2)	98.1 (59.6–180.4)	0.05
Vasoactive substances			
Angiopoietin-1 (ng/mL)	3.27 (2.01–5.36)	2.97 (1.42–5.15)	0.1
Angiopoietin-2 (ng/mL)	9.51 (5.06–13.2)	5.14 (3.04–11.18)	< 0.0001*
sTie2 (ng/mL)	16.03 (10.91–19.51)	8.36 (6.67–12.85)	< 0.0001*

Values are shown as median (interquartile range)

*AaDO*_*2*_ alveolar-arterial oxygen difference, *CRP* C-reactive protein, *EVLWI* extravascular lung water index, *GEDI* global end-diastolic index, *HCO*_*3*_^*−*^ arterial bicarbonate concentration, *HR* heart rate, *IL* interleukin, *INR* international normalized ratio, *MAP* mean arterial pressure, *NE* norepinephrine, *pCO*_*2*_ arterial partial pressure of carbon dioxide, *PCT* procalcitonin, *PLT* platelet count, *sTie2* soluble receptor of tyrosine kinase with immunoglobulin-like and EGF-like domains 2, *SVRI* systemic vascular resistance index, *SVV* stroke volume variance, *WBC* white blood cell count

*Significant *p* values

### Effects of TPE on biochemical parameters, circulating cytokines, and vasoactive substances

Humoral markers of inflammation were elevated in all patients, but did not change after TPE. Besides a reduction in cytokines known for their involvement in the pathophysiology of sepsis (e.g., IL-1b 147.1 (57.1–241.6) vs. 92.2 (42.9–184.8) pg/mL, *p* = 0.01; Table 2), we also observed reductive effects on permeability-inducing factors such as angiopoietin-2 (9.5 (5.1–13.2) vs. 5.1 (3.1–11.2) ng/mL, *p* < 0.0001). On the other hand, the antipermeability factor angiopoietin-1 (3.27 (2.01–5.36) vs. 2.97 (1.42–5.15) ng/mL, *p* = 0.1) was unchanged after TPE. The anti-inflammatory cytokine IL-10 (143.3 (65.5–259.2) vs. 98.1 (59.6–180.4) pg/mL, *p* = 0.05) was slightly reduced after TPE, although this did not reach statistical significance (Table 2).

In addition, we observed an improved acid-base balance, although continuous RRT that had been started in 65% of the patients before TPE was discontinued during the time of TPE (pH 7.28 (7.19–7.34) vs. 7.33 (7.23–7.38), *p* = 0.01).

### Predictors of responsiveness to TPE

To identify potential predictors of TPE responsiveness, we defined two types of responses: immediate (reduction of the NE dose > 20%) and sustained (any reduction in SOFA score within 48 h) in accord with the literature [16]. Fifty percent of patients (10/20) were immediate responders and 35% (7/20) were sustained responders. Subgroup analyses in each group were performed for numerous baseline characteristics (Additional file 3: Table S2). However, multivariable regression analysis could not identify independent predictors of acute or sustained TPE responsiveness.

### Effect on 28-day mortality

**Fig. 3:**
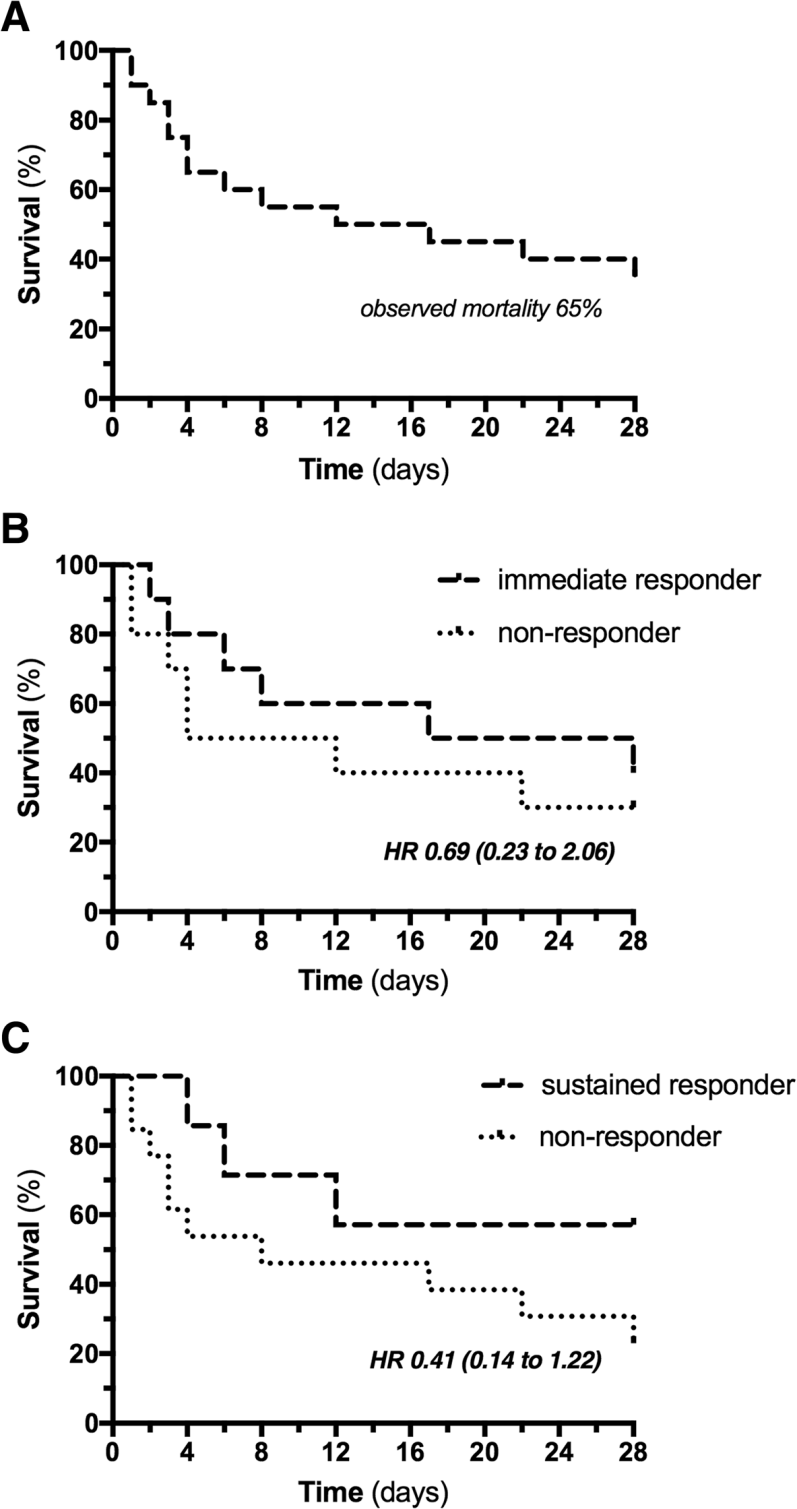
Twenty-eight-day survival. Kaplan Meier graphs showing the 28-day survival course in **a** the overall cohort showing an observed mortality of 65%, **b** immediate responders (*n* = 10) and nonresponders (*n* = 10) to plasma exchange (defined as norepinephrine reduction of > 20%), as well as **c** sustained responders (*n* = 7) and nonresponders (*n* = 13) to plasma exchange (defined as any reduction in SOFA score within 48 h following plasma exchange). HR hazard ratio

The observed 28-day mortality was 65% (Fig. 3a). Median 28-day survival was 14.5 days. In the “immediate-responder” group, mortality was 60% and median 28-day survival was 22.5 days; mortality in the “nonresponder” group was 70% and median 28-day survival 8 days (HR (hazard ratio) 0.69; 95% CI 0.23 to 2.06; *p* = 0.38; Fig. 3b). In the “sustained-responder” group, mortality was 43% and median 28-day survival was 28 days; mortality in the “nonresponder” group was 77% and median 28-day survival was 8 days (RR 0.41; 95% CI 0.14 to 1.22; *p* = 0.137; Fig. 3c).

### Effect on vascular barrier function: ex-vivo analysis

**Fig. 4:**
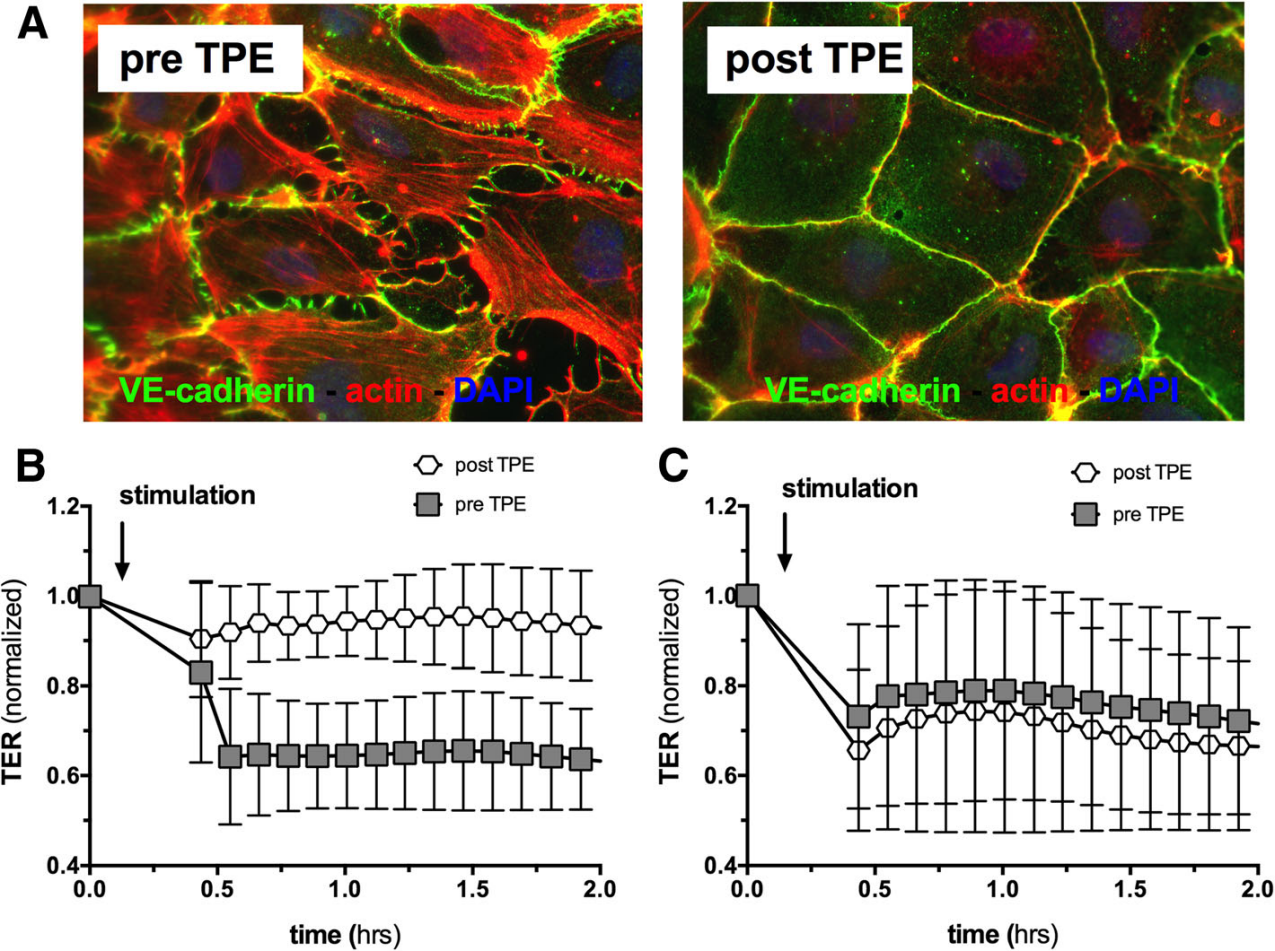
Ex-vivo effect of plasma obtained from patients with septic shock on endothelial morphology and function. **a** HUVECs were incubated for 30 min with patients plasma obtained immediately before (left panel) and after (right panel) therapeutic plasma exchange (TPE) ex vivo. Immunofluorescent cytochemistry for the cell-cell contact protein VE-cadherin (green) and the cytoskeletal component f-actin (red) show severe alterations of the endothelial architecture and the formation of paracellular gaps (i.e., the cellular correlate of the clinical capillary leakage syndrome). Incubation of HUVECs with the same patients plasma obtained after TPE did not induce these changes any more. This assay was performed with plasma from all patients. Shown are images from a representative patient. **b** Transendothelial electrical resistance (TER), a highly quantitative method to assess permeability in real time in vitro, revealed that 60% (12/20) of patients plasma did induce a severe drop in resistance (grey dots). The same patients plasma after TPE did not induce permeability any more (white bars). **c** 40% (8/20) of patients did not show any response to therapeutic TPE with regard to TER before and after the procedure

We exposed HUVECs to plasma before and after TPE and assessed their phenotype by fluorescent immunocytochemistry. Cell-cell contacts were analyzed by the adherens junction protein VE-cadherin (green) whereas the cytoskeletal architecture was visualized by F-actin (red). Septic shock plasma induced the formation of focal adhesions, actin stress fibers, and multiple paracellular gaps, changes not observed when the experiment was performed with plasma that was obtained from the same patient, but 15 min after TPE (Fig. 4a). We additionally performed a quantitative functional assay by measuring the TER (i.e., the permeability) in real time. This method revealed that 60.0% of patients’ plasma showed improvements (Fig. 4b) whereas 40% of patients’ plasma showed no change in its permeability-inducing capacity (Fig. 4c). We grouped the patients according to their response in the ECIS assay and found that the mortality in the ECIS response group was 58.4% whereas it was 75% in the ECIS nonresponsive group.

## Discussion

This prospective, nonrandomized, single-center explorative study examined the feasibility and preliminary efficacy of TPE as an additive treatment strategy in septic shock. In summary, we found the following:

- feasibility and safety of the procedure
- hemodynamic improvement indicated by a NE reduction often achieved within minutes after TPE start
- improved preload and fluid balance possibly due to a protective effect on vascular permeability
- decline in plasma concentrations of proinflammatory mediators
- reversibility of the septic endothelial phenotype ex vivo from 60% of patients plasma after TPE

A recent meta-analysis found four single-center RCTs that analyzed TPE in sepsis [12]. In adults, TPE was associated with a reduced mortality. The largest of those trials (*n* = 106 patients) showed an encouraging trend towards improved survival (33.3% vs. 53.8%) [13]. Unfortunately, this study was underpowered and included a heterogeneous group of patients in terms of disease severity (< 60% with shock) and time of onset.

We believe that both timing and disease severity might be crucial for a beneficial effect of TPE. Therefore, we exclusively included patients that met the strict criteria with regard to onset (i.e. < 12 h) and severity (i.e. NE > 0.4 μg/kg/min) of shock. Before initiation of this study, we have occasionally treated sepsis patients with a particularly severe shock with TPE as rescue therapy. We routinely performed three TPEs on consecutive days but realized that hemodynamic improvements were only seen after the first (not the second or the third) treatment.

Under prospective study conditions, we could now collect data that might support our earlier encouraging observations. Ten out of 20 patients showed an immediate response, determined as a reduction of vasopressor > 20% and four patients showed a reduction of > 50%; one patient was even completely weaned off any vasopressors at the end of TPE. In 7 of 20 patients, an improvement in organ failure indicated by a SOFA score reduction was achieved within the first 48 h following TPE and this sustained response was also associated with a trend towards better survival. Unfortunately, we were not able to identify predictors of TPE responsiveness in this small cohort. Obviously, this study was neither designed nor powered to address the effects of TPE on survival. However, 65% of our patients died, underscoring both disease severity and also the fact that TPE does not provide a cure for septic shock, but potentially an adjunctive therapeutic option with beneficial effects.

A major difference between TPE and modern extracorporeal adsorption strategies [20] is based on the fact that the exchange of septic shock plasma with FFP might not lead to an unselective depletion of pro- and anti-inflammatory cytokines. It rather replenishes protective factors (within the FFPs) that had been consumed by the sepsis. Given the role of cytokines in the physiological host response to a local infection, a complete depletion of cytokines might not necessarily be beneficial per se. The exchange of septic against healthy plasma might be a procedure by which these circulating factors can be modulated but not completely removed. This hypothesis is supported by our findings with regard to the anti-permeability factor angiopoietin-1.

The beneficial effect of TPE on preload and fluid balance might reflect improved vascular barrier function. Alternatively, it is possible that the observed rapid hemodynamic stabilization was due to oncotic effects of the relatively large amount of FFPs that was substituted within 2 h during TPE. However, our cell culture studies are in line with the permeability hypothesis, as we found fewer endothelial alterations if the cells were challenged with plasma after TPE compared with the individual plasma before TPE.

This study has important limitations, mainly its small sample size, the single-center setting, and its nonrandomized nature. Given the lack of a control group, all positive effects observed during the course of TPE could have been unrelated to the intervention. In addition, the intervention was administered at a fixed dose, which precludes us from providing data on effects at different dosages or time frames. At inclusion to the study, 95% of patients were sedated and endotracheally intubated for mechanical ventilation. Their Glasgow Coma Score (GCS) at inclusion was therefore (artificially) determined to be 3 points. Using a GCS calculated at admission to the ICU (before sedation) would have yielded lower APACHE II scores. Unfortunately, we do not have this information from all patients and we wanted to reflect the baseline status at the inclusion time point. The NE cut-off of 0.4 μg/kg/min for inclusion is truly arbitrary and might not be optimal. The average NE doses of septic shock patients in many large-scale international RCTs is lower than our chosen 0.4 μg/kg/min (e.g., [21–24]). However, some studies have also reported higher baseline NE requirements in septic shock [25]. The ideal NE dose to include the sickest septic shock patients has yet to be determined.

This explorative study was not designed to assess survival but to determine the feasibility and safety of a larger RCT and to assess preliminary efficacy. Given that we were able to enroll 20 patients within a year, we believe that such a multicenter RCT addressing clinical outcomes appears feasible.

## Conclusions

Our exploratory study demonstrated preliminary safety and feasibility of TPE in early septic shock patients and we are currently preparing a randomized, controlled, multicenter study to further assess this treatment.

## Additional files

Additional file 1: **Table S1.** Microbial spectrum and initial anti-infective therapy. Demonstrated are characteristics of the site of infection, infectious pathogen species, initial anti-infective regimen, and sensitivity of the pathogen to initial therapy for each patient. (DOCX 106 kb)

Additional file 2: **Figure S1.** Hemodynamics assessed by thermodilution. Box and whisker blots showing extended hemodynamics assessed by thermodilution technique (PiCCO®, Pulsion) before (pre-) and after (post-) plasma exchange. The grey areas in all graphs highlight the reference range in healthy individuals. Assessment of (A) myocardial performance by the cardiac index (CI), (B) afterload by the systemic vascular resistance index (SVRI), and preload by (C) global end-diastolic volume index (GEDI) and (D) the dynamic stroke volume variance (SVV). (E) Vascular permeability was analyzed by the extravascular lung water index (EVLWI). (TIFF 388 kb)

Additional file 3: **Table S2.** Possible determinants of immediate and sustained clinical response to plasma exchange. Compared are differences in clinical and biochemical characteristics for the subgroups immediate response/nonresponse and sustained response/nonresponse, respectively. (DOCX 23 kb)

## Abbreviations

APACHE: Acute Physiology and Chronic Health Evaluation
CBA: Cytometric bead array
CI: Cardiac index
CRP: C-reactive protein
ECIS: Electric cell-substrate impedance sensing
FACS: Fluorescence-activated cell sorting
FFP: Fresh frozen plasma
GEDI: Global end-diastolic volume index
HUVEC: Human umbilical vein endothelial cell
ICU: Intensive care unit
IL: Interleukin
INR: International normalized ratio
MAP: Mean arterial pressure
NE: Norepinephrine
PCT: Procalcitonin
RCT: Randomized controlled trial
RRT: Renal replacement therapy
SOFA: Sequential Organ Failure Assessment
sTie2: Soluble receptor of tyrosine kinase with immunoglobulin-like and EGF-like domains 2
SVRI: Systemic vascular resistance index
SVV: Stroke volume variance
TER: Transendothelial electrical resistance
TPE: Therapeutic plasma exchange
VEGF: Vascular endothelial growth factor
vWF: von-Willebrand factor
WBC: White blood cell
