# Supplementary material for: Early therapeutic plasma exchange in septic shock: a prospective open-label nonrandomized pilot study focusing on safety, hemodynamics, vascular barrier function, and biologic markers
Source: Crit Care. 2018 Oct 30;22:285. doi: 10.1186/s13054-018-2220-9 (PMC6206942; doi:10.1186/s13054-018-2220-9)
Supplement: Supplementary file 1 — Table S1. Microbial spectrum and initial anti-infective therapy. Demonstrated are characteristics of the site of infection, infectious pathogen species, initial anti-infective regimen, and sensitivity of the pathogen to initial therapy for each patient. (DOCX 106 kb) [file 13054_2018_2220_MOESM1_ESM.docx]

ABBREVIATIONS:

BC blood culture, BAL bronchoalveolar lavage
