# Supplementary figures and images for: Early therapeutic plasma exchange in septic shock: a prospective open-label nonrandomized pilot study focusing on safety, hemodynamics, vascular barrier function, and biologic markers
Source: Crit Care. 2018 Oct 30;22:285. doi: 10.1186/s13054-018-2220-9 (PMC6206942; doi:10.1186/s13054-018-2220-9)

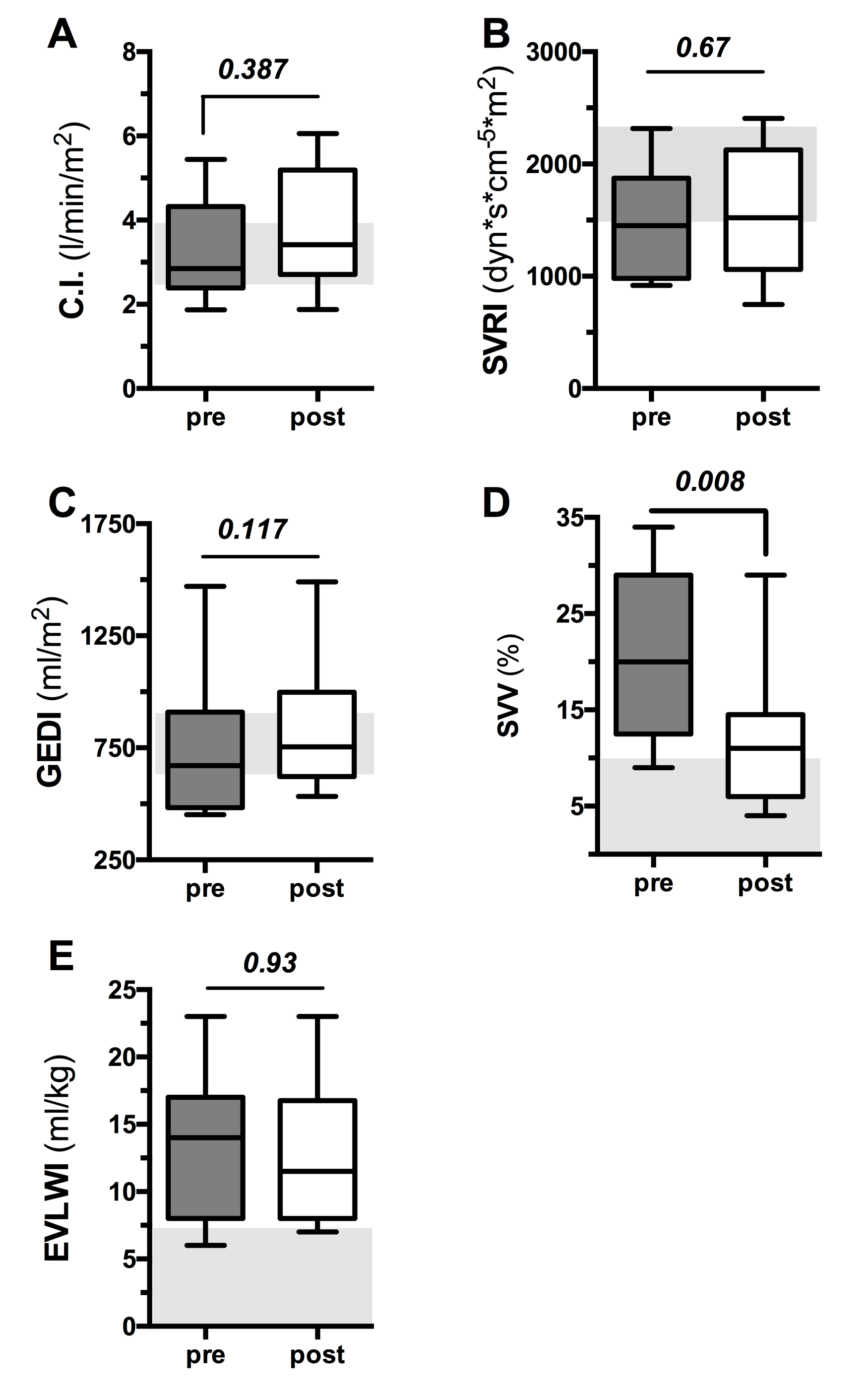

Supplement: Supplementary file 2 — Figure S1. Hemodynamics assessed by thermodilution. Box and whisker blots showing extended hemodynamics assessed by thermodilution technique (PiCCO®, Pulsion) before (pre-) and after (post-) plasma exchange. The grey areas in all graphs highlight the reference range in healthy individuals. Assessment of (A) myocardial performance by the cardiac index (CI), (B) afterload by the systemic vascular resistance index (SVRI), and preload by (C) global end-diastolic volume index (GEDI) and (D) the dynamic stroke volume variance (SVV). (E) Vascular permeability was analyzed by the extravascular lung water index (EVLWI). (TIFF 388 kb) [file 13054_2018_2220_MOESM2_ESM.tiff]
