# Supplementary material for: Early therapeutic plasma exchange in septic shock: a prospective open-label nonrandomized pilot study focusing on safety, hemodynamics, vascular barrier function, and biologic markers
Source: Crit Care. 2018 Oct 30;22:285. doi: 10.1186/s13054-018-2220-9 (PMC6206942; doi:10.1186/s13054-018-2220-9)
Supplement: Supplementary file 3 — Table S2. Possible determinants of immediate and sustained clinical response to plasma exchange. Compared are differences in clinical and biochemical characteristics for the subgroups immediate response/nonresponse and sustained response/nonresponse, respectively. (DOCX 23 kb) [file 13054_2018_2220_MOESM3_ESM.docx]

| **Table S2:** Possible determinants of immediate and sustained clinical response to plasma exchange. | | | | | | |  |
| --- | --- | --- | --- | --- | --- | --- | --- |
| ***Characteristic*** | ***Immediate*** | | ***p*** | ***Sustained*** | | ***p*** |  |
|  | ***Responder*** *(n=10)* | ***Non-Responder*** *(n=10)* |  | ***Responder*** *(n=7)* | ***Non-Responder*** *(n=13)* |  |  |
| Site of infection - no (%) |  |  |  |  |  |  | |
| pulmo | 4 (40) | 8 (80) | ns | 4 (57) | 7 (54) | ns | |
| abdomen | 3 (30) | 1 (10) | ns | 0 (0) | 3 (23) | ns | |
| urogenital | 1 (10) | 0 (0) | ns | 1 (14) | 0 (0) | ns | |
| soft tissue | 2 (20) | 1 (10) | ns | 1 (14) | 2 (15) | ns | |
| endocarditis | 1 (10) | 0 (0) | ns | 1 (14) | 0 (0) | ns | |
| identified pathogen - no (%) |  |  |  |  |  |  | |
| gram + | 0 (0) | 2 (20) | ns | 1 (14) | 1 (8) | ns | |
| gram - | 5 (50) | 1 (10) | ns | 2 (29) | 4 (31) | ns | |
| fungi | 1 (10) | 1 (10) | ns | 0 (0) | 2 (14) | ns | |
| viral | 0 (0) | 1 (10) | ns | 0 (0) | 1 (8) | ns | |
| mixed | 2 (20) | 2 (20) | ns | 0 (0) | 4 (31) | ns | |
| Non-identified | 2 (20) | 3 (30) | ns | 4 (57) | 1 (8) | p=0.015 | |
| initial APACHE II | 35.5 (30.5-42.25) | 42 (35.75-47.25) | ns | 36 (26-48) | 41 (35-44.5) | ns | |
| initial SOFA | 17 (14.25-19.25) | 19.5 (17.5-22) | ns | 20 (18-22) | 17 (15.5-19.5) | ns | |
| Norepinephrine dose (ug/kg/min) | 0.85 (0.55-1.12) | 0.82 (0.62-1.58) | ns | 0.67 (0-61-0.88) | 1.00 (0.61-1.71) | ns | |
| Mechanical ventilation - no (%) |  |  |  |  |  |  | |
| yes | 9 (90) | 10 (100) | ns | 6 (86) | 13 (100) | ns | |
| no | 1 (10) | 0 (0) | ns | 1 (14) | 0 (0) | ns | |
| Oxygenation-index (PaO_2_/FiO_2_) | 126 (84-258) | 133 (113-211) | ns | 195 (87-317) | 129 (99-162) | ns | |
| Renal replacement therapy - no (%) | 4 (40) | 6 (60) | ns | 5 (71) | 8 (62) | ns | |
| Organ failure - no (%) |  |  |  |  |  |  | |
| respiratory | 9 (90) | 10 (100) | ns | 6 (86) | 13 (100) | ns | |
| coagulation | 7 (70) | 7 (70) | ns | 6 (86) | 8 (62) | ns | |
| liver | 5 (50) | 5 (50) | ns | 6 (86) | 4 (31) | p=0.019 | |
| cardiovascular | 10 (100) | 10 (100) | ns | 7 (100) | 13 (100) | ns | |
| neurological | 9 (90) | 10 (100) | ns | 6 (86) | 13 (100) | ns | |
| renal | 6 (60) | 10 (100) | p=0.025 | 6 (86) | 10 (77) | ns | |
| Multi organ failure (MOV) - no (%) |  |  |  |  |  |  | |
| two | 0 (0) | 0 (0) | ns | 0 (0) | 0 (0) | ns | |
| three | 2 (20) | 0 (0) | ns | 1 (14) | 0 (0) | ns | |
| four | 3 (30) | 2 (20) | ns | 0 (0) | 6 (47) | p=0.032 | |
| five | 4 (40) | 4 (40) | ns | 2 (29) | 5 (38) | ns | |
| six | 1 (10) | 4 (40) | ns | 4 (57) | 2 (15) | ns | |
| Immunosuppression - no (%) |  |  |  |  |  |  | |
| yes | 7 (70) | 6 (60) | ns | 4 (57) | 10 (77) | ns | |
| no | 3 (30) | 4 (40) | ns | 3 (43) | 3 (23) | ns | |
| Leucocytosis - *1000/ul | 11.2 (0.3-30.8) | 11.6 (2.4-37.7) | ns | 9.0 (0.9-15.7) | 18.4 (0.65-43.4) | ns | |
| CRP - mg/l | 228.5 (142.8-380) | 238 (131.5-294) | ns | 221 (147-286) | 237 (135-351) | ns | |
| PCT - ug/l | 24.1 (18-87.5) | 34.1 (5.2-91.3) | ns | 23.8 (17.3-122.5) | 28.7 (4.0-98.6) | ns | |
| Angpt-2 ng/ml | 9.5 (5.5-14.5) | 9.8 (4.9-13.8) | ns | 10.3 (6.1-14.9) | 9.5 (4.8-14.4) | ns | |
| sTie2 | 18.7 (12.6-21.5) | 14.5 (10.5-18.9) | ns | 18.6 (14.4-19.5) | 15.7 (10.5-21.7) | ns | |
| IL-6 | 6.4 (1.9-18.2) | 17.2 (2.8-35.4) | ns | 19.4 (1.4-28.2) | 8.7 (2.6-29.5) | ns | |
| IL-1b | 105.9 (49.5-235.2) | 179.6 (61.2-230.6) | ns | 166.2 (45.9-225 | 128 (59.2-366.4) | ns | |
| ADAMTS-13 - % | 40.5 (28-61.5) | 55 (29-71) | ns | 51 (32.75-76) | 42 (25-55) | ns | |
| Side of infection - no (%) |  |  |  |  |  |  | |
| pulmo | 4 (40) | 8 (80) | ns | 4 (57) | 7 (54) | ns | |

ABBREVIATIONS:

SOFA - Sequential Organ Failure Assessment, APACHE - Acute Physiology And Chronic Health Evaluation, CRP – C-reactive protein, PCT – procalcitonine, IL – interleukin, ADAMTS13 - A disintegrin and metalloprotease with thrombospondin-1-like domains 13
